# Beyond MIND and Mediterranean Diets: Designing a Diet to Optimize Parkinson’s Disease Outcomes

**DOI:** 10.3390/nu17142330

**Published:** 2025-07-16

**Authors:** Laurie K. Mischley, Magdalena Murawska

**Affiliations:** 1 Bastyr University Research Institute, Bastyr University, Kenmore, WA 98028, USA; 2 Department of Radiology, School of Medicine, University of Washington, Seattle, WA 98109-8057, USA; 3 Parkinson Center for Pragmatic Research, Shoreline, WA 98133, USA; mmurawska@biostatcode.org

**Keywords:** nutrition, lifestyle modification, clinical epidemiology, positive deviance, neurodegenerative

## Abstract

**Background**: A growing body of evidence suggests that diet can modify Parkinson’s disease (PD) outcomes, although there is disagreement about what should be included and excluded in such a diet. Existing evidence suggests that adherence to the MIND and Mediterranean (MEDI) diets are associated with reduced PD symptoms, but only a few variables from the adherence scales are responsible for the statistically observed improvement. **Objectives**: The goal was to use patient-reported outcomes in a large cohort to identify the foods and dietary patterns (PRO diet) most strongly associated with the fewest PD symptoms over time, and to develop a composite adherence scale to enable comparisons between MEDI, MIND, and PRO. **Methods**: Data were obtained from the prospective longitudinal natural history study and from Modifiable Variables in Parkinsonism (MVP)—a study designed to identify behaviors associated with patient-reported outcomes (PRO-PD). Upon the completion of the binary and food frequency data collection, using various predictive models and considering congruence with historical data, the PRO diet was created via an iterative process. Our goal was to create a new scale and compare its performance to the existing MIND and MEDI scores. The comparison was made at baseline, using the regression models for PRO-PD and the different scales as the predictors. The models were compared via the Akaike Information Criterion (AIC). To examine whether baseline adherence levels predicted subsequent symptom trajectories, the baseline PRO diet adherence and subsequent slope of progression were evaluated. **Results**: Data from 2290 individuals with PD were available for this analysis. The Mediterranean and MIND diets showed almost identical effects. For both the diets, the effect they had on non-motor symptoms was about twice the effect on motor symptoms. The slopes for the total PRO-PD for MEDI, MIND, and PRO-21 were −64.20467, −64.04220, and −28.61995, respectively. The AIC value differences were substantial (>2), indicating meaningful improvements in the model fit for total PRO-PD, as follows: MEDI: 28,897.24, MIND: 28,793.08, and PRO-21: 27,500.71. The subset of individuals who were most adherent to the PRO-21 diet at baseline had the slowest subsequent progression, as measured by a 43% reduced PRO-PD slope, compared to the less adherent groups. **Conclusions**: The PRO-21 outperformed the MIND and MEDI diets in the model fit, overcoming the ceiling effects and showing orders of magnitude and superior explanatory power for variance in PD outcomes, despite the smaller per-unit effect sizes. However, its rigorous demands may introduce barriers related to cost, feasibility, and sustainability, underscoring the need for future intervention trials to assess real-world feasibility, adherence, side effects, and clinical impact.

## 1. Introduction

Dietary interventions are of great interest because of their potential to modify the symptoms and trajectory of Parkinson’s disease (PD). Almost twenty years ago, large prospective epidemiological studies began to demonstrate that prudent dietary patterns—rich in fruit, vegetables, and fish—were inversely associated with PD risk, whereas Western dietary patterns were associated with an increased risk of a subsequent PD diagnosis [1,2]. While pesticide exposure and dairy intake are among the most well-established risk factors for PD incidence [3], not a single intervention study has attempted an organic, dairy-free diet. With a consistent and growing body of observational evidence suggesting that diet has the potential to impact the risk of PD and PD progression, intervention studies aiming to use diet as a disease-modifying strategy in PD are warranted. Thus far, the most studied approaches include the Mediterranean (MEDI) and MIND (Mediterranean–DASH Intervention for Neurodegenerative Delay) diets. While each has some evidence of potential benefit [4,5,6,7], none of these diets were explicitly designed or optimized for PD.

Based on observations in Crete and southern Italy, the MEDI diet was first formally described by Ancel Keys in 1970 as being associated with lower rates of coronary heart disease [8]. As the diet is modified to fit geographic and cultural habits, globally, the retained value of the modified diet and the sustainability of these MEDI-based diets has been called into question. To overcome this barrier, researchers have codified the diet via a MEDI adherence screener (MEDAS) tool to aid research [9,10]. A recent meta-analysis found a significant negative correlation between MEDI dietary adherence and the subsequent risk of PD, particularly of prodromal features [11]. A small randomized controlled trial suggested that MEDI diets improved fiber intake and reduced intestinal inflammation in people with PD, although the improvement in bowel function was not superior to the standard of care [12]. A 10-week randomized controlled trial of the MEDI diet compared to a control demonstrated that MEDI adherence “remarkably increased the dimensions of executive function, language, attention, concentration, and active memory…” [13]. A randomized controlled trial comparing the traditional Iranian diet to the MEDI diet also demonstrated an improvement in beta carotene, selenium, and the total antioxidant capacity of the blood, as well as a reduction in the caloric energy intake in those randomized to the MEDI arm. After the 10-week study, participants in the MEDI arm had significant reductions in all components of the UPRDS, except for motor.

The MIND diet was developed by combining elements of the MEDI and DASH diets, and was designed for cognitive health [14]. As opposed to the MEDI diet, the MIND emphasis is on berries, leafy greens, and limited butter and cheese. In a cross-sectional analysis of a Canadian cohort, MIND adherence was associated with a later PD diagnosis age [15]. A US-based longitudinal study demonstrated that greater MIND adherence was associated with a decreased risk of parkinsonism and a slower rate of progression [16]. Despite the MIND diet’s seeming superiority in PD outcomes over the MEDI diet [6,16], it incorporates poultry, permits some dairy, and makes no mention of dietary pesticide exposure, meaning that it is out of alignment with Parkinson-specific research.

While MEDI and MIND diets are well-defined and have available strategies for addressing adherence, none have been optimized for PD; they were developed with other primary health goals in mind and were only later tested in PD populations. Most concerning, individual dietary components within these diets are at odds with PD-specific research (e.g., they include dairy), and only a handful of variables drive most of the observed effects. With the MEDI diet, sofrito was associated with worse PRO-PD scores over time, although this was not statistically significant. Only the following four variables were statistically associated with improved outcomes: less than one sweet or carbonated beverage per day; three or more servings of nuts per week; less than one serving per day of red meat; and two or more vegetable servings per day, including one raw or as a salad. In the MIND diet, however, the inclusion of poultry was associated with non-statistically significant worse PRO-PD outcomes, with the following variables meeting statistical significance: frequently eating nuts, green leafy vegetables, other vegetables, and berries, and avoiding cheese, red meat, butter, and margarine [6]. While associated with a trend toward benefit, in neither of the studies were legumes, wine, or olive oil independently associated with outcomes. These data suggest the MEDI and MIND diets may have more value to people with PD if they did not include poultry and sofrito, respectively.

While the MEDI and MIND diets have demonstrated promising associations with improved PD outcomes, neither was explicitly developed or optimized for PD populations. Notably, these diets include components that conflict with PD-specific research and only a subset of their variables appear to drive most of the observed benefits. To address this gap, the present work introduced the PRO diet, a curated set of dietary criteria that were derived from the MVP dataset and designed to optimize patient-reported outcomes. This composite adherence scale offers a targeted tool for patients, providers, and researchers seeking to advance nutritional strategies that meaningfully impact PD’s trajectory.

## 2. Materials and Methods

Inspired by the positive deviance research model, the MVP study was designed as a pragmatic, prospective, and internet-based observational study, designed to describe the behaviors and habits associated with patient-centered symptoms of severity. With the protected time and support afforded by the National Institutes of Health K01-ATT004404; the Career Transition Award; the didactic training in patient-centered outcomes; and nutritional and neuro-epidemiology support from the Bastyr University and the University of Washington School of Public Health, respectively, the MVP project was designed to be a purely pragmatic and patient-centered approach for identifying the positive deviants with PD. The study has sent out two surveys per year to the study participants. Participants are recruited online, during lectures, at local support groups, and at conferences, where possible. Attempts have been made to reach underserved communities, including rural America and others globally. Detailed recruitment methods have been described in prior publications. A wide variety of incomes are represented, although this cohort is disproportionately white and highly educated [17,18,19,20]. To date, there are 3763 individuals being followed longitudinally in the MVP Study, inclusive of individuals with prodromal symptoms of parkinsonism and Parkinson-plus syndromes. This dataset only includes individuals who enrolled before the end of 2023, provided informed consent, and reported a diagnosis of idiopathic PD.

The primary outcome measures of PD severity and progression in the MVP study are the patient-reported outcomes in PD (PRO-PD), a validated, continuous outcome measure, designed to quantify global symptom severity in individuals with PD. The PRO-PD quantifies the subjective user experience across 33 common motor and non-motor PD symptoms. All symptoms are rated on a slider from 0 (not present) to 100 (severe). The total PRO-PD score is the sum of these individual symptom scores, yielding a composite score ranging from 0 to 3300, with higher scores indicating a greater overall symptom burden. Unlike traditional clinician-administered rating scales, the PRO-PD is self-reported and emphasizes non-motor symptoms that frequently precede motor decline, making it especially useful for capturing early disease progression.

The goal was to develop a diet based on optimized PRO-PD scores, which lacks the ceiling effect seen in MIND and MEDI diets, has all variables individually associated with better PD outcomes, and provides a path to fewer PD symptoms compared to the MEDI and MIND diet adherence. To derive the dietary pattern most strongly associated with slowed PD progression, we conducted a comprehensive, data-driven analysis of all the available food frequency and binary dietary variables in the MVP dataset. The variables were grouped and tested to evaluate the dose–response relationships with symptom trajectories, using PRO-PD scores as the primary outcome. Variables were retained based on their individual and cumulative predictive power for slower symptom accumulation over time. This modeling process ultimately yielded a 21-point adherence scale—the PRO-21 diet score—comprising the foods and dietary behaviors most consistently associated with favorable long-term outcomes. The scoring system reflects both the inclusion and exclusion of items associated with faster progression.

**Figure 1:**
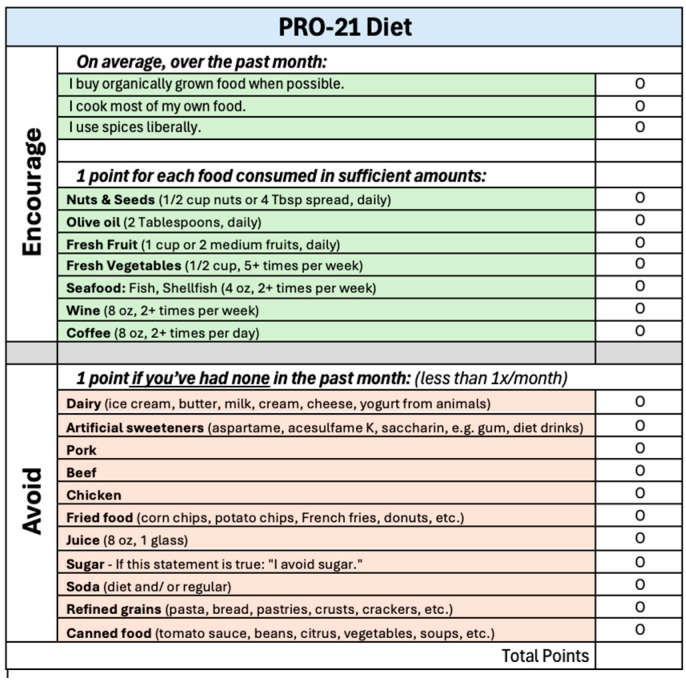
Sample patient handout or clinical training tool.

The final 21-variable PRO-21 diet score was developed based on the components most strongly and consistently associated with slower progression on the PRO-PD scale. The score awards one point for each of the following 10 positive behaviors: consuming adequate amounts of nuts and seeds (½ cup or 2 Tbsp of spread per day); olive oil (≥1 Tbsp/day); fresh fruit (≥1 cup/day); fresh vegetables (≥1½ cups/day); seafood (fish or shellfish ≥ 2 times/week); red wine (≥2 servings/week); coffee (≥2 cups/day); the liberal use of spices; cooking at home; and buying organically grown food. Additional points were awarded for complete or near-complete avoidance (i.e., less than once per month) of the following 11 items: dairy (from animal sources); pork; beef; chicken; fried food; soda (regular and diet); refined grains (bread and pasta); juice; sugar; artificial sweeteners (e.g., aspartame, acesulfame K, and saccharin); and eating from a can (Figure 1). The final model suggests that the complete avoidance of pro-inflammatory and ultra-processed items, rather than moderation, was most predictive of better outcomes. As such, the PRO-21 diet represents a prescriptive data-derived eating pattern, with the goal of informing future intervention trials aimed at modifying PD progression.

To test the validity of the newly established PRO diet, the MIND, MEDI, and PRO diet scores were computed and compared at baseline. The subsequent slope of progression was plotted based on the baseline scores. Only individuals with a diagnosis of idiopathic PD (N = 2290) were used in this analysis; individuals reporting Parkinson-plus syndromes or other forms of parkinsonism were dropped from the analysis. Baseline distributions were illustrated and each score was modeled using linear regression models, adjusting for age, sex, income, and years since diagnosis.

The validated MIND and MEDI [14,21] dietary adherence scores were incorporated into the MVP study to make the MIND and MEDI adherence scores available for the study participants. The PRO diet is a Parkinson-specific dietary model derived from PRO-PD scores. As new data and perspectives emerge, variations of this model are anticipated. The current version, PRO-21, includes 21 variables and represents the best-fitting model in this analysis.

The Akaike Information Criterion (AIC) model was used to compare how well the different statistical models fitted the dataset. It balances the goodness of fit and simplicity, with a lower AIC, meaning a better balance between fit and simplicity. To validate the model’s performance, AIC values across the MEDI, MIND, and PRO dietary adherence scores were compared.

Cross-validation (CV) was conducted to evaluate the generalizability of each model beyond the development dataset. While the AIC assesses model fit within a given sample, it does not account for potential overfitting or generalizability, or for predictive performance on new data. CV estimates model stability across multiple simulated subsamples. Robust error metrics—the Root Median Squared Error (RMdSE) and Median Absolute Error (MedAE)—were selected to minimize the influence of outliers. CV was performed using 100 randomly generated subsamples, each comprising 30% of the dataset.

A univariate analysis was conducted to examine the associations between the individual PRO diet score components and PD outcomes, unadjusted and adjusted for age, gender, income, and years since diagnosis. All statistical analyses were performed using R version 4.2.1 and RStudio version 2024.12.0.

## 3. Results

**Figure 2:**
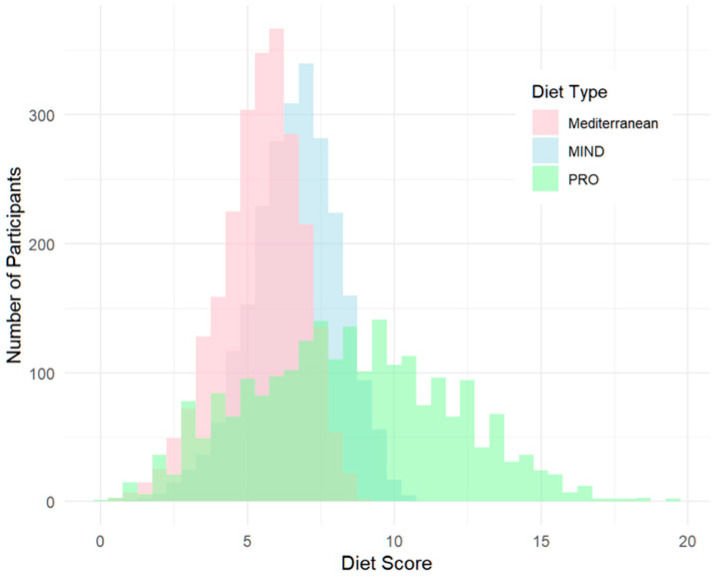
Adherence to Mediterranean, MIND, and PRO diets in a cohort of people with PD (N = 2290).

Of the 2788 participants enrolled in the study, 2290 reporting a diagnosis of PD were included in the analysis (Table 1). The histogram (Figure 2) illustrates that the PRO diet has a broader, more even distribution, extending to higher adherence levels without an apparent ceiling effect. Favorably, the histogram suggests that the PRO-21 diet allows for greater differentiation among highly adherent individuals, offering a wider measurement range and potentially capturing meaningful variance, which is saturated or undetectable in the MIND and MEDI frameworks. Psychologically, there are disadvantages to this broader distribution and high ceiling—with only 109/2290 (4.8%) of people scoring above 15, the bar for excellence may seem unreachable or overwhelming.

The models tested whether any of the diets were associated with PRO-PD scores after adjusting for age, gender, income, and years since diagnosis. In all three models, dietary adherence demonstrated a significant inverse association with PRO-PD outcomes (MEDI: β = −64, 95% CI −78 to −51, *p* < 0.001; MIND: β = −64, 95% CI −77 to −51, *p* < 0.001; PRO-21: β = −29, 95% CI −34 to −23, *p* < 0.001), indicating that higher adherence was associated with a lower symptom burden. Importantly, the number of years since diagnosis was consistently and positively associated with PRO-PD scores (β ≈ 28–29, *p* < 0.001), reflecting the expected worsening over time. Income also showed a robust inverse association, with lower income predicting worse outcomes across all models (β ≈ −41 to −43, *p* < 0.001). Notably, neither age nor gender showed significant independent effects in the fully adjusted models (age: *p* > 0.7; gender: *p* ≈ 0.1–0.4). All three diet scores showed strong significant associations with PRO-PD outcomes (*p* < 0.001), suggesting that the better adherence to any of these diets is linked to better PRO-PD scores (Table 2).

**Figure 3:**
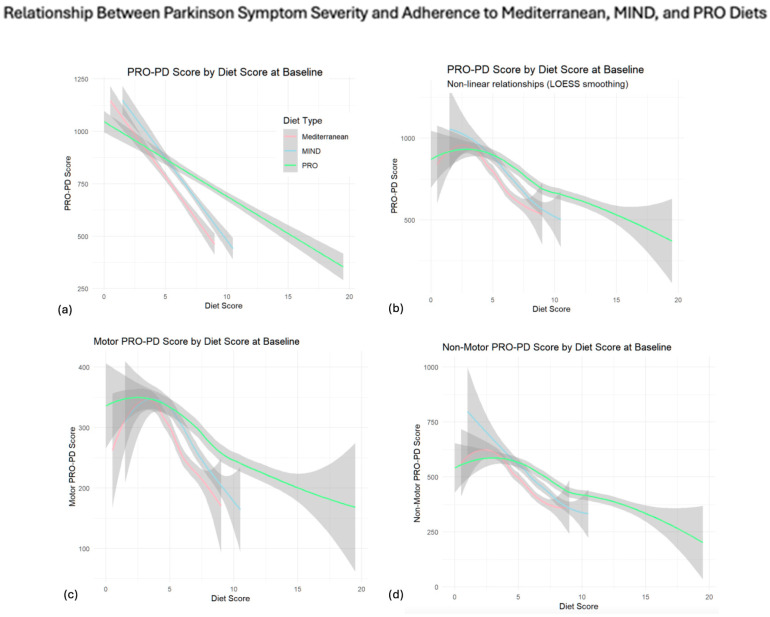
Linear models (**a**), LOESS smoothing for total PRO-PD scores (**b**), motor sub-scores (**c**), and non-motor sub-scores (**d**).

Figure 3 illustrates both the linear and non-linear relationships between the dietary adherence scores and the PRO-PD outcomes at baseline. The first figure shows that the fitted linear models (Figure 3a–d) use LOESS smoothing to expose the non-linear dynamics. For the total PRO-PD scores, strict adherence to the MEDI and MIND scores were associated with a maximum reduction in the PRO-PD score, ~500 (good), whereas the PRO-21 diet scores above 15, which were rare, were associated with even lower PRO-PD scores, below 500, which have been associated with “excellent” quality of life [22]. While the MEDI and MIND scores showed larger per-unit reductions in symptom burden (−21 to −22 points/unit for motor; −43 to −44 points/unit for non-motor), the PRO-21 score maintained a consistent, statistically significant effect across both domains (−10 points/unit motor; −18 points/unit non-motor), despite its expanded adherence range. Importantly, non-motor outcomes appeared to be more sensitive to dietary effects overall, with larger beta estimates across all models.

The PRO-21 diet model demonstrated an improved statistical fit relative to the MEDI and MIND diet models, with AIC values that were 1397 and 1282 points lower, respectively (Table 3). While AIC differences greater than two are generally considered meaningful, these substantially larger deltas indicated that, among the models tested, the PRO-21 score offered superior explanatory power for PRO-PD symptom burden. Notably, this improvement in model fit was achieved despite smaller per-unit effect sizes, suggesting that the PRO-21 score may capture relevant variance in symptom outcomes through a broader dietary signal. These findings reflect model-based predictive performance and do not imply causality or validate specific dietary components.

**Figure 4:**
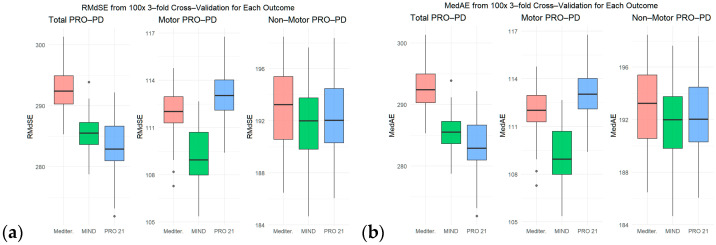
Cross-validation analysis across diet models. (**a**) Root mean square error (RMdSE) from 100 iterations of 3-fold cross-validation, where lower values indicate better model fit. (**b**) Median absolute error (MedAE) from the same runs, with lower values indicating more accurate and reliable predictions. Mediterranean (Mediter.).

The cross-validation results (Figure 4a, RMdSE; Figure 4b, MedAE) show that, while the PRO-21 diet consistently achieved the lowest error values across the total and non-motor PRO-PD outcomes, the MIND diet outperformed both the MEDI and PRO-21 models for motor PRO-PD, exhibiting the lowest RMdSE and MedAE in this domain. This suggests that, although PRO-21 offers the best overall model fit and predictive accuracy, the MIND diet may capture motor symptom variance more effectively, highlighting nuanced differences in how each dietary pattern relates to specific symptom domains. These findings underscore the importance of considering both overall and domain-specific performance when evaluating dietary models.

**Figure 5:**
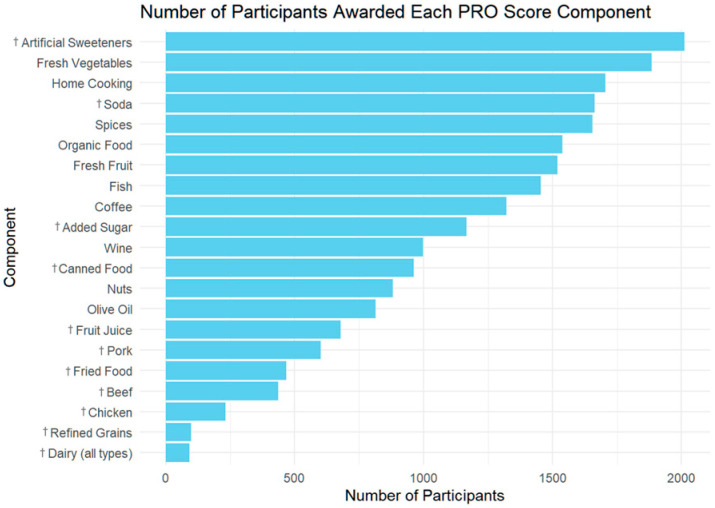
Participant adherence to individual PRO-21 score components. Points were awarded for avoiding variables marked with †.

**Figure 6:**
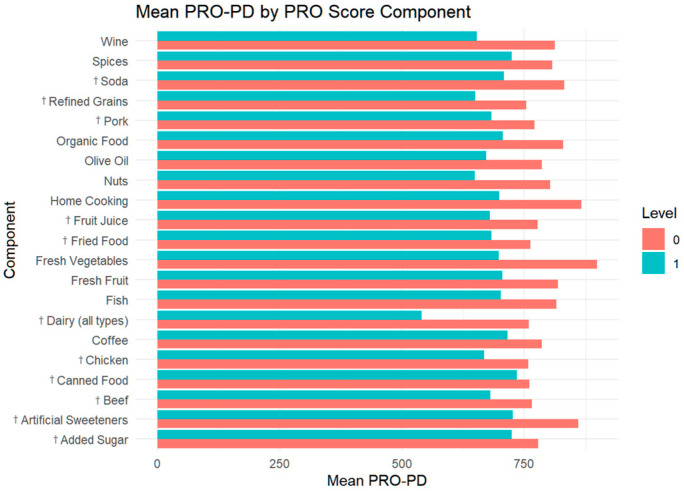
Mean symptom severity stratified by PRO-21 component adherence. PRO-PD scores of individuals that did and did not receive the point for each of the variables comprising the PRO-21 diet. Points were awarded for avoiding variables marked with †.

The data show that dairy abstention was the least commonly achieved PRO component, with only a small number of participants receiving this point (Figure 5). Canned food was the variable least associated with differences in PD symptom severity. Despite its rarity, dairy abstainers exhibited a substantial difference in their mean PRO-PD scores compared to dairy consumers, with a lower symptom burden observed among those avoiding dairy (Figure 6). Similarly, few participants were awarded points for avoiding refined grains or consuming nuts, though these components also showed notable differences in the mean PRO-PD scores among the groups. In contrast, components such as avoiding sweeteners, consuming fresh vegetables, and cooking at home were among the most frequently achieved behaviors, with relatively small mean PRO-PD score differences among the groups.

**Figure 7:**
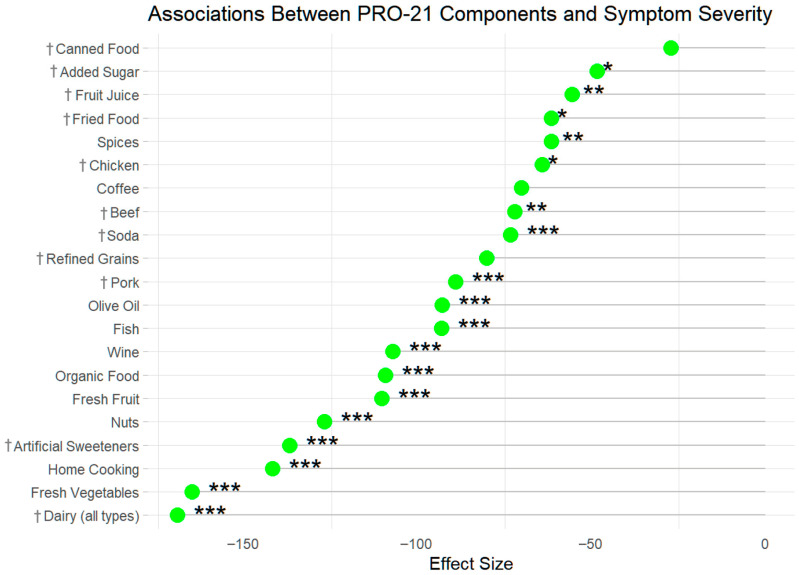
Ranked impact of PRO-21 components by effect size and significance levels. †—points were awarded if these variables were avoided. * *p* < 0.05, ** *p* < 0.01, *** *p* < 0.000.

In adjusted models, all PRO-21 components were independently associated with lower PRO-PD symptom scores, an a priori requirement for inclusion in the diet. The largest absolute estimates were observed among the participants with a high adherence to vegetable intake, home cooking, and dairy avoidance. While most components retained statistician significance after adjustment, refined grains, pork, and canned food showed attenuated associations, indicating variability in the strength of individual dietary predictors. These results suggest that certain dietary behaviors may be more strongly aligned with symptom burden than others, even within an overall adherence framework (Table 4; Figure 7).

### PRO-21 Dietary Adherence and Subsequent Slope of Progression

**Figure 8:**
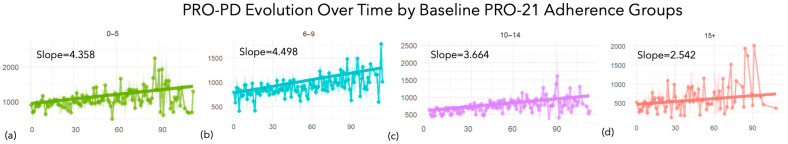
Baseline PRO-21 dietary adherence and subsequent evolution of Parkinson’s symptoms. (**a**) PRO-21 score 0–5, (**b**) PRO-21 score 6–9, (**c**) PRO-21 score 10–14, (**d**) PRO-21 score 15+. Patient-reported outcomes (PRO-PD scores) were used to measure symptom severity over time. All slopes: *p* < 0.0001.

To evaluate whether adherence to PRO-21 was related to the subsequent rate of PD progression, time slope estimates were assigned to four categories of adherence: 0–5, 6–9, 10–14, and 15+ (Table 5; Figure 8). The highest level of adherence, 15 points or more, while rare (n = 109, 4.8%), was associated with a statistically significant 45% reduction in the subsequent slope of progression for up to 100 months of observation. These analyses adjusted for age, gender, and income, but did not consider any dietary changes that participants may have made after baseline reporting.

**Figure 9:**
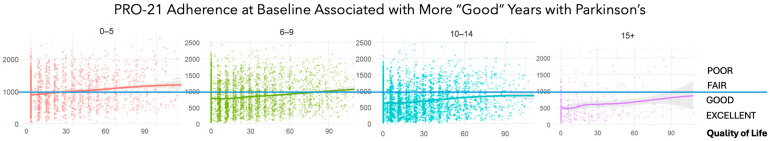
Baseline PRO-21 dietary adherence and subsequent evolution of Parkinson symptoms. PD symptoms were measured with the patient-reported outcomes in PD (PRO-PD) and quality of life thresholds based on prior publications: 0–500—excellent; 500–1000—good; 1000–1500—fair; 1500–2000—poor. The horizontal blue line (PRO-PD = 1000) marks the boundary between “Good” and “Fair” for quality of life, showing that higher dietary adherence is associated with more years spent with “Good” quality of life.

Because statistical significance does not necessarily equate to clinical relevance, Figure 9 illustrates that individuals in the lower PRO-21 adherence cohorts exhibited greater symptom burden at baseline. Encouraging adherence to the PRO-21 dietary pattern among individuals with a prodromal parkinsonism diet may be a strategy for pre-emptive symptom mitigation.

## 4. Discussion

Across all the dietary models, years since diagnosis, income, and diet were significant predictors of PRO-PD outcomes, while age and gender did not impact the results. Importantly, non-motor outcomes appeared more sensitive to dietary effects overall, with larger beta estimates across all models. While the MIND diet may offer short-term symptomatic benefit (Figure 3), the PRO-21 model was associated with more favorable long-term trajectories, suggesting that it may be more well-suited for sustained symptom mitigation over time. The MEDI and MIND diets clustered heavily between 0 and 10, creating a ceiling effect that artificially compressed the apparent dietary impact, whereas the PRO-21 score had a wider span, allowing differentiation among highly adherent individuals and revealing potential dose–response relationships beyond the limits of the other two scales. While the MEDI and MIND adherence curves flattened at moderate levels (suggesting diminishing returns), the PRO-21 curve continued downward, capturing a more gradual and sustained association with improved outcomes across its extended range. Together, these results suggest that the PRO-21 score offers not only statistical advantages in terms of distribution and variance capture, but also clinical relevance by distinguishing gradations of adherence that are unable to be resolved using MEDI and MIND tools.

Over the past three decades, dairy intake has emerged as a consistent dietary factor associated with increased PD risk [3,23]. Although a minority of participants in this cohort avoided dairy entirely, those who did had substantially lower mean PRO-PD scores (Figure 4 and Figure 6), suggesting a potentially meaningful clinical impact. Among all PRO-21 components, dairy avoidance demonstrated one of the strongest associations with reduced symptom burden. Although no study has definitively determined whether dairy avoidance alters the course of PD after diagnosis, the consistency of associations observed across studies [24,25,26]—and now of symptom severity—warrants thoughtful consideration as to if, when, and how to encourage patients to adhere to a dairy-free diet. What may be empowering to one person may be overwhelming to another. Not only is dairy avoidance commonly contested by patients—out of convenience and preference—the recommendation to avoid dairy for the sake of preventing PD progression may create new stressors for individuals in assisted care facilities or facing challenges related to nutritional access.

The PRO diet model was stronger when two glasses of wine per week and two cups of coffee per day were included, which was congruent with a large body of traditional epidemiological data demonstrating that coffee and alcohol are associated with a decreased risk of diagnosis [27,28]. In coffee, numerous biologically active molecules, especially caffeine and phenolic compounds, have been proposed to play a protective role [29,30,31]. Two recent meta-analyses reported an inverse association between alcohol consumption and PD risk, and there has been an over-representation of non-drinkers among PD patients [32,33]. For reasons ranging from alcoholism to taste aversion, many may find these findings inconvenient, or even repugnant, including providers and researchers who are increasingly recognizing the negative physiological and societal impacts of alcohol. Both coffee and red wine contain volatile compounds with known or suspected antioxidant properties, and it is biologically plausible that some volatile components enter the brain via the olfactory bulb. Just as inhaled toxicants have been proposed as etiological agents in PD [34,35,36], so might inhaled volatile nutrients from coffee and wine offer neuroprotection [37].

The liberal intake of olive oil and nuts is also congruent with traditional epidemiological data in PD [1,38] and they are common across the MEDI, MIND, and PRO-21 diets. The Spanish PREDIMED study demonstrated that both a MEDI diet supplemented with extra virgin olive oil and a MEDI diet supplemented with mixed nuts resulted in an approximate 30% reduction in the risk of CVD [39], and it is exciting to anticipate similar studies in PD.

**Figure 10:**
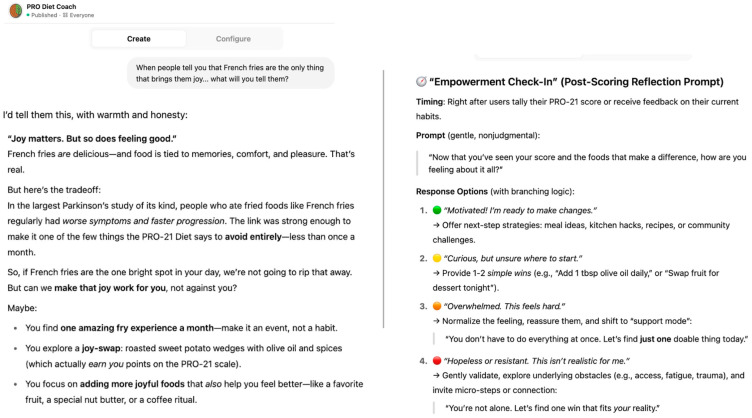
Example of a nutritional coaching system that takes patient psychological status into consideration. Branch logic used to personalize guidance and engage user appropriately.

As evidence linking diet to PD risk and symptom burden continues to accumulate, building accessible, evidence-informed dietary guidance for both patients and providers will be essential to ensure that recommendations are scientifically grounded but also practical, personalized, and supportive of long-term adherence. Dietary training programs and online resources for patients and providers are likely to be developed over time, as the demand is recognized. Figure 10 shows screenshots from the PRO Diet Coach, a GPT-based conversational tool that was developed to deliver personalized, psychologically informed dietary coaching. Based on users’ responses, the tool has been programmed to use branching logic to adapt messaging [40].

Patients should be taught that all variables are associated with improvement, and they should start with what feels most attainable. While PRO-21 adherence scores above 15 surpassed what the MIND and MEDI diets seemed capable of statistically, fewer than 5% of participants reached that threshold. Replacing dairy-based butter with olive oil, adding cinnamon and apple to oatmeal, and eating celery dipped in peanut butter are examples of easy, affordable strategies to gain additional points. Nutritional medicine practitioners are encouraged to develop handouts (Figure 1), courses, and educational events designed to normalize whole-food, plant-based, home-cooked organic meals, abundant in nuts, olive oil, spices, fresh fruits and vegetables, seafood, coffee, and wine. Whole grains, such as brown rice, quinoa, barley, legumes, and eggs were neutral and should be encouraged for satiety, fiber, and substance.

## 5. Conclusions

The PRO-21 diet consistently outperformed the MIND and MEDI diets in the model fit, as indicated by its substantially lower AIC values across motor, non-motor, and total PD symptoms. This superior statistical fit emerged despite PRO-21 showing smaller per-symptom effect sizes, suggesting it captures meaningful variance in disease outcomes beyond the ceiling effects observed in MIND and MEDI adherence scores. However, PRO-21’s rigorous adherence demands may introduce new barriers, including increased patient frustration, stress, and feasibility concerns, both for individuals and providers. Achieving high PRO-21 adherence (scores of 15+) may come with challenges related to cost, accessibility, dietary satisfaction, and long-term sustainability, which have yet to be studied. Intervention trials are necessary to test the feasibility, adherence potential, side effects (including the financial and psychological burdens), and real-world impact of PRO-21 compared to the MIND and MEDI diets.

## Figures and Tables

**Table 1:** Participant characteristics.

		Number of PRO-21 Points at Baseline				
Participant Characteristics	All Participants	0–5 Points	6–9 Points	10–14 Points	15+ Points	p.overall
	N = 2290	n = 454	n = 893	n = 832	n = 111	
Age	64.1 [57.1;69.7]	63.0 [54.7;69.5]	64.0 [57.3;69.6]	64.7 [58.1;69.6]	63.4 [55.0;70.4]	0.021
Gender (n = 2277)						.
Male	988 (43.4%)	262 (58.1%)	391 (44.0%)	289 (34.9%)	46 (41.8%)	
Female	1288 (56.6%)	189 (41.9%)	498 (56.0%)	537 (64.9%)	64 (58.2%)	
Non-binary	1 (0.04%)	0 (0.00%)	0 (0.00%)	1 (0.12%)	0 (0.00%)	
Income (n = 2163)						0.004
Less than $20,000	122 (5.64%)	28 (6.53%)	39 (4.63%)	51 (6.47%)	4 (3.85%)	
Between $20–40,000	258 (11.9%)	62 (14.5%)	105 (12.5%)	83 (10.5%)	8 (7.69%)	
Between $40–60,000	287 (13.3%)	68 (15.9%)	109 (12.9%)	99 (12.6%)	11 (10.6%)	
Between $60–80,000	295 (13.6%)	60 (14.0%)	128 (15.2%)	97 (12.3%)	10 (9.62%)	
Between $80–100,000	304 (14.1%)	65 (15.2%)	131 (15.6%)	95 (12.1%)	13 (12.5%)	
Between $100–150,000	417 (19.3%)	68 (15.9%)	162 (19.2%)	159 (20.2%)	28 (26.9%)	
More than $150,000	480 (22.2%)	78 (18.2%)	168 (20.0%)	204 (25.9%)	30 (28.8%)	
Geographic Location: (n = 1316)						.
Africa	9 (0.68%)	2 (0.95%)	4 (0.84%)	3 (0.55%)	0 (0.00%)	
Asia	10 (0.76%)	1 (0.48%)	1 (0.21%)	8 (1.46%)	0 (0.00%)	
Europe	197 (15.0%)	23 (11.0%)	61 (12.9%)	94 (17.2%)	19 (22.4%)	
North America	1035 (78.6%)	174 (82.9%)	380 (80.2%)	416 (76.1%)	65 (76.5%)	
Oceania	59 (4.48%)	7 (3.33%)	28 (5.91%)	23 (4.20%)	1 (1.18%)	
South America	6 (0.46%)	3 (1.43%)	0 (0.00%)	3 (0.55%)	0 (0.00%)	
Estimated Hoehn & Yahr, by patient (n = 2245)						.
1-sided symptoms only, minimal disability	1266 (56.4%)	198 (44.9%)	473 (53.9%)	524 (64.1%)	71 (64.5%)	
Both sides affected, balance is stable	395 (17.6%)	87 (19.7%)	140 (16.0%)	147 (18.0%)	21 (19.1%)	
Mild to moderate disability, balance affected	508 (22.6%)	130 (29.5%)	235 (26.8%)	127 (15.5%)	16 (14.5%)	
Severe disability, able to walk and stand without help	48 (2.14%)	17 (3.85%)	18 (2.05%)	12 (1.47%)	1 (0.91%)	
Confinement to bed or wheelchair unless aided	4 (0.18%)	2 (0.45%)	1 (0.11%)	1 (0.12%)	0 (0.00%)	
Don’t know	24 (1.07%)	7 (1.59%)	10 (1.14%)	6 (0.73%)	1 (0.91%)	
Years since diagnosis, at baseline (n = 2211)	3.21 [1.29;6.48]	4.08 [1.67;8.05]	3.21 [1.39;6.63]	2.93 [1.15;5.54]	2.47 [0.90;5.22]	<0.001
PRO-PD Symptom severity score, mean (n = 2074)	647 [374;1050]	881 [537;1235]	713 [415;1126]	534 [330;872]	396 [257;704]	<0.001
PRO-21 Diet score, mean (n = 2290)	8.50 [6.00;11.0]	4.00 [3.00;4.50]	7.50 [6.50;8.50]	11.0 [10.0;12.5]	15.0 [14.5;16.0]	0

**Table 2:** Relative impact of Mediterranean, MIND, and PRO diets on Parkinson symptom severity. CI—confidence interval.

Impact of Dietary Adherence on Patient-Reported Outcomes in Parkinson’s Disease									
	Impact on Motor Symptoms			Impact on Non-Motor Symptoms			Impact on Total PRO-PD Score		
	Beta	95% CI	*p*-Value	Beta	95% CI	*p*-Value	Beta	95% CI	*p*-Value
Mediterranean	−21	−27, −16	<0.001	−44	−53, −35	<0.001	−64	−78, −51	<0.001
Age	0.12	−0.71, 0.94	0.8	−0.59	−1.9, 0.76	0.4	−0.14	−2.2, 1.9	0.9
Sex, Female	−19	−34, −4.3	0.012	−13	−37, 12	0.3	−33	−71, 5.2	0.091
Income	−15	−19, −11	<0.001	−28	−34, −21	<0.001	−41	−51, −31	<0.001
Years since diagnosis	14	13, 16	<0.001	15	12, 17	<0.001	29	25, 33	<0.001
MIND	−22	−27, −17	<0.001	−43	−51, −35	<0.001	−64	−77, −51	<0.001
Age	0.34	−0.48, 1.2	0.4	−0.28	−1.6, 1.1	0.7	0.38	−1.7, 2.4	0.7
Sex, Female	−19	−34, −4.1	0.013	−11	−36, 13	0.4	−32	−69, 5.9	0.1
Income	−15	−19, −11	<0.001	−28	−34, −21	<0.001	−41	−51, −31	<0.001
Years since diagnosis	14	13, 16	<0.001	15	12, 17	<0.001	29	25, 33	<0.001
PRO-21	−10	−12, −7.9	<0.001	−18	−22, −15	<0.001	−29	−34, −23	<0.001
Age	0.29	−0.54, 1.1	0.5	−0.49	−1.9, 0.88	0.5	0.11	−2.0, 2.2	>0.9
Sex, Female	−15	−30, 0.93	0.065	−2.3	−28, 23	0.9	−18	−57, 21	0.4
Income	−16	−20, −12	<0.001	−30	−37, −23	<0.001	−43	−53, −33	<0.001
Years since diagnosis	14	13, 16	<0.001	14	12, 17	<0.001	28	24, 33	<0.001

**Table 3:** Model fit and symptom trajectory differences across Mediterranean, MIND, and PRO-21 diets.

Diet	Symptom Type	Slope (β)	Per-Symptom Effect	AIC
Mediterranean	Motor	−21.49	−1.65	26,227.41
	Non-Motor	−43.84	−2.19	27,980.33
	Total	−64.20	—	28,897.24
MIND	Motor	−21.90	−1.68	26,118.01
	Non-Motor	−42.80	−2.14	27,901.36
	Total	−64.04	—	28,793.08
PRO	Motor	−10.09	−0.77	24,861.99
	Non-Motor	−18.44	−0.92	26,646.29
	Total	−28.62	—	27,500.71

Effect size and model performance metrics by diet type: motor, non-motor, and total PRO-PD scores.

**Table 4:** Independent effects of PRO-21 diet components on symptom severity in PD (N = 2788).

		Unadjusted				Adjusted		
	Estimate	StdError	*t*-Value	*p*-Value	Estimate	StdError	*t*-Value	*p*-Value
Dairy–less than 1 serving per month	−219.81	49.477	−4.443	0	−169.612	48.609	−3.489	0
Fresh vegetables (1/2 cup, 5+ times per week)	−200.868	21.54	−9.325	0	−165.335	21.636	−7.642	0
I cook most of my own food.	−168.009	19.903	−8.442	0	−142.123	21.09	−6.739	0
Artificial sweeteners–“I avoid artificial sweeteners.”	−134.451	23.283	−5.775	0	−137.153	23.147	−5.925	0
Nuts & seeds (1/2 cup nuts or 4 Tbsp spread, daily)	−153.387	19.87	−7.72	0	−127.209	19.729	−6.448	0
Fresh fruit (1 cup or 2 medium fruits, daily)	−114.03	19.44	−5.866	0	−110.626	19.411	−5.699	0
I buy organically grown food when possible.	−122.869	19.321	−6.359	0	−109.593	19.197	−5.709	0
Wine (8 oz, 2+ times per week)	−159.021	19.263	−8.255	0	−107.394	19.49	−5.51	0
Seafood: fish, shellfish (4 oz, 2+ times per week)	−113.378	19.267	−5.885	0	−93.317	19.325	−4.829	0
Olive oil (2 Tablespoons, daily)	−113.576	20.307	−5.593	0	−93.201	20.154	−4.624	0
Pork–less than 1 serving per month	−89.052	22.35	−3.985	0	−89.336	22.172	−4.029	0
Refined grains (pasta, bread)–less than 1 per month	−103.921	50.236	−2.069	0.039	−80.398	49.496	−1.624	0.104
Soda (diet and/or regular)–less than 1 serving per month	−124.051	20.145	−6.158	0	−73.538	20.415	−3.602	0
Beef–less than 1 serving per month	−84.884	24.996	−3.396	0.001	−72.184	24.812	−2.909	0.004
Coffee (8 oz, 2+ times per day)	−140.295	38.312	−3.662	0	−70.401	38.186	−1.844	0.065
Chicken–less than 1 serving per month	−90.441	32.847	−2.753	0.006	−64.326	32.791	−1.962	0.05
I use spices liberally.	−83.371	20.005	−4.167	0	−61.746	20.031	−3.083	0.002
Fried food–less than 1 serving per month	−79.371	24.586	−3.228	0.001	−61.728	24.139	−2.557	0.011
Juice (8 oz, 1 glass)–less than 1 serving per month	−98.348	21.406	−4.594	0	−55.721	21.502	−2.591	0.01
Sugar–“I avoid sugar.”	−54.92	19.106	−2.875	0.004	−48.578	19.005	−2.556	0.011
Canned food–less than 1 serving per month	−24.548	19.846	−1.237	0.216	−27.243	19.707	−1.382	0.167

Component-level associations driving PRO-21 score relationship with PRO-PD outcomes.

**Table 5:** Baseline adherence to PRO-21 and subsequent slope of PD progression.

Linear Unadjusted Mixed Model Results by PRO-21 Group						
PRO-21 Adherence Cohort	Time Slope	Std. Error	*t* Value	*p*-Value	Observations	n
0–5 points	4.451	0.23	19.38	0	1795	448
6–9 points	4.48	0.157	28.62	0	3748	879
10–14 points	3.691	0.179	20.63	0	3450	818
15+ points	2.889	0.484	5.96	0	383	109

## Data Availability

The data are not publicly available due to institutional ownership and ethical considerations. The dataset, which includes over 10 years of longitudinal information on more than 3500 individuals, is owned by Bastyr University and contains sensitive health information. To ensure responsible use and participant confidentiality, de-identified data subsets may be made available to qualified researchers upon request and under a formal licensing agreement with Bastyr University.
